# Je, a versatile suite to handle multiplexed NGS libraries with unique molecular identifiers

**DOI:** 10.1186/s12859-016-1284-2

**Published:** 2016-10-08

**Authors:** Charles Girardot, Jelle Scholtalbers, Sajoscha Sauer, Shu-Yi Su, Eileen E.M. Furlong

**Affiliations:** European Molecular Biology Laboratory, Genome Biology Unit, Heidelberg, D-69117 Germany

**Keywords:** Software, Genomics, NGS, UMI, Multiplexing, Duplicates

## Abstract

**Background:**

The yield obtained from next generation sequencers has increased almost exponentially in recent years, making sample multiplexing common practice. While barcodes (known sequences of fixed length) primarily encode the sample identity of sequenced DNA fragments, barcodes made of random sequences (Unique Molecular Identifier or UMIs) are often used to distinguish between PCR duplicates and transcript abundance in, for example, single-cell RNA sequencing (scRNA-seq). In paired-end sequencing, different barcodes can be inserted at each fragment end to either increase the number of multiplexed samples in the library or to use one of the barcodes as UMI. Alternatively, UMIs can be combined with the sample barcodes into composite barcodes, or with standard Illumina® indexing. Subsequent analysis must take read duplicates and sample identity into account, by identifying UMIs.

**Results:**

Existing tools do not support these complex barcoding configurations and custom code development is frequently required. Here, we present Je, a suite of tools that accommodates complex barcoding strategies, extracts UMIs and filters read duplicates taking UMIs into account. Using Je on publicly available scRNA-seq and iCLIP data containing UMIs, the number of unique reads increased by up to 36 %, compared to when UMIs are ignored.

**Conclusions:**

Je is implemented in JAVA and uses the Picard API. Code, executables and documentation are freely available at http://gbcs.embl.de/Je. Je can also be easily installed in Galaxy through the Galaxy toolshed.

**Electronic supplementary material:**

The online version of this article (doi:10.1186/s12859-016-1284-2) contains supplementary material, which is available to authorized users.

## Background

High-throughput sequencing has become the approach of choice in genomic experiments (RNA-seq, ChIP-seq, DNA-seq, …). Continuous improvements in sequencing chemistry and hardware have translated into significant cost decreases with huge increases in productivity (up to 400 million reads per lane on current Illumina® HiSeq 4000). This throughput often exceeds the sequencing depth required in many applications [1], or when working with small genomes. Protocols to sequence multiple samples within the same sequencer lane (multiplexed library) are now common practice in both single end (SE) and paired end (PE) strategies. Multiplexing can also be guided by experimental design considerations where samples are sequenced in different lanes to gain information on technical variance, or in staged sequencing approaches to reduce sequencing costs where samples are sequenced sequentially until the required sequencing depth is achieved [1].

**Fig. 1:**
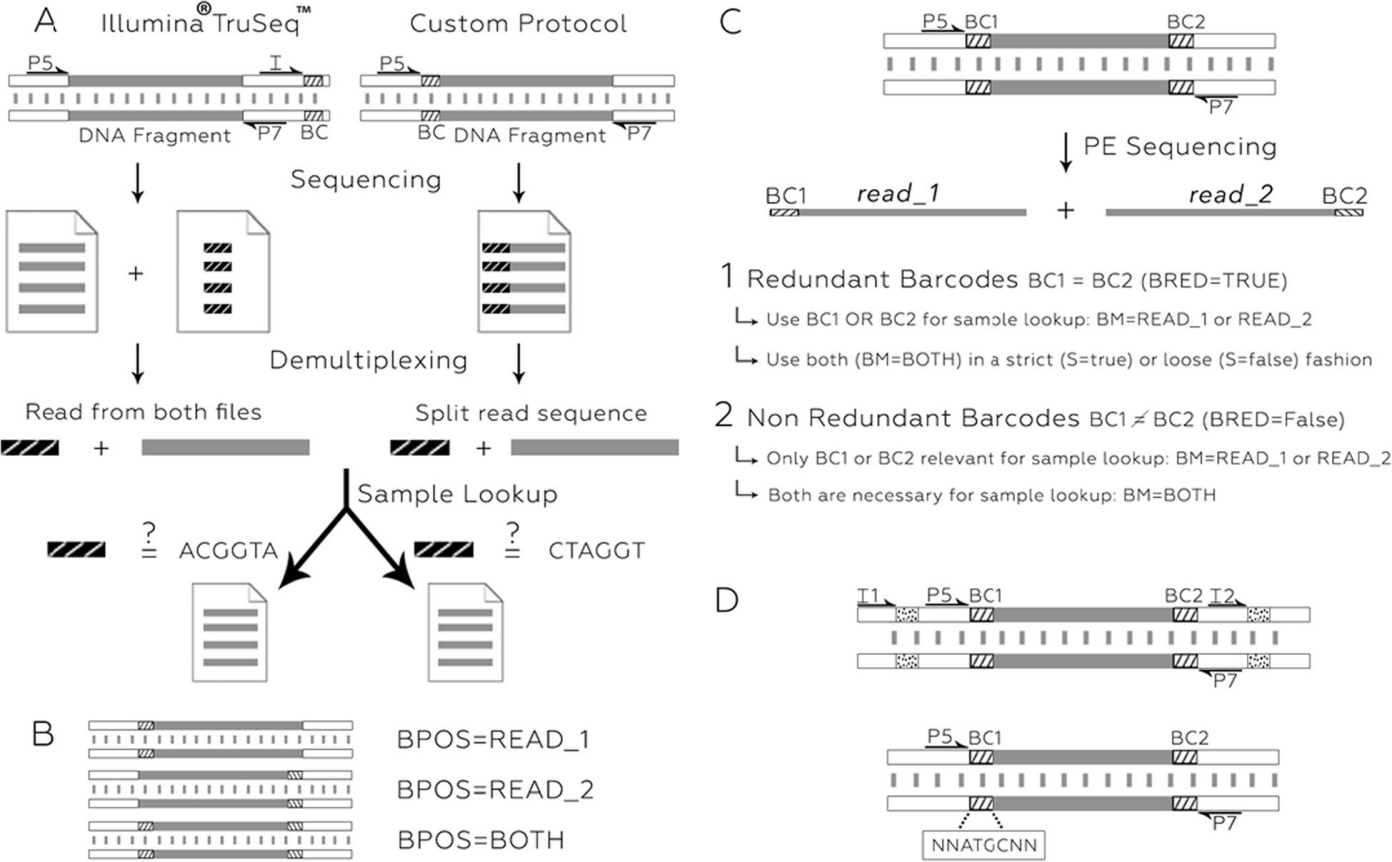
Barcoding Strategies. **a** Schematic view of the multiplexed library processing. A unique and different barcode (BC, white box with black stripes) is used for each sample. The barcode is placed further down the DNA fragment and sequenced in a specific sequencing round (Illumina® TruSeq™, left); or directly upstream the DNA fragment and sequenced concomitantly (custom protocol, right). After sequencing and image processing, reads of multiplexed samples are mixed together in the fastq result file. For each read, the barcoding sequence (black box with white stripes) is computationally clipped off the read end (custom protocols) or read from the additional barcode file (Illumina® TruSeq™, index file is provided with the I1 option); and the original sample is identified by comparing this barcoding sequence to known barcodes. Finally, read sequences are saved in sample specific fastq files. **b** In PE sequencing, barcodes can be added to one or both fragment ends. The Je *demultiplex* BPOS option indicates which read(s) contain(s) the barcode(s). **c** *demultiplex* options for barcodes present at both read ends. A decision is needed to specify which barcode is used to identify separate samples. **d** Combining UMIs (BC1 and BC2, white box with black stripes) with Illumina sample indexing (white box with black dots, top) or as composite barcode (bottom). In a composite barcode, the number of random base upstream and downstream the sample index is variable

In multiplexed libraries, DNA fragments originating from the same sample are associated with a unique sequence of fixed length (e.g. six bases). This barcode (or index) is later used to computationally identify the original sample of each sequenced read. In the Illumina® TruSeq™ protocol (Fig. 1a, left), the barcode is inserted further down the DNA fragment and debarcoding is usually performed using the Illumina CASAVA pipeline. In alternative protocols (Fig. 1b, right), the barcode is inserted directly upstream of the DNA fragment during library construction and the debarcoding operation is typically performed using third party tools [2, 3] (also see Additional file 1: Table S1 for features comparison) or custom code.

Custom multiplexing protocols offer great design flexibility, in particular in PE sequencing where barcodes can be inserted at one or both ends of the DNA fragment (Fig. 1b). In the latter, the barcode found in each read of the pair is usually the same, and this redundancy allows for more specificity when one of the barcoding sequences contains errors or bases of poor quality. The encoding possibilities are exponentiated by adapting a different barcode to each end of the DNA fragment. Lastly, the correct interpretation of experiments, such as single cell RNA-seq (scRNA-seq), requires the disentanglement of biological read duplicates that reflect RNA abundance in the cell from technical duplicates that result from sequencing the same RNA molecule multiple times (PCR duplicates). A common procedure towards this goal is to barcode each DNA fragments before PCR amplification i.e. each read is attached to a fixed-length (random) sequence that will act as a Unique Molecular Identifier (UMI) [4–7]. After read mapping, only duplicate reads with different UMIs will be kept in downstream processing. UMIs can be combined with sample barcodes in different ways, which varies between protocols: using separate ends of the DNA fragments (Fig. 1c, case 2), combining Illumina sample indexing with custom barcoding to add a UMI to DNA fragment ends (Fig. 1d, top) or using composite barcodes (Fig. 1d, bottom).

**Fig. 2:**
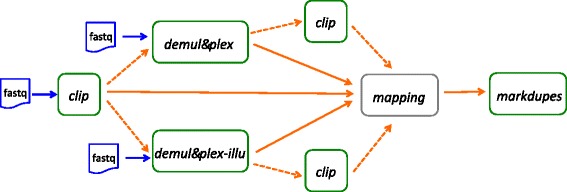
The different modules of Je (green squared blocks) and their usage in workflows. The *clip*, *demultiplex* and *demulitplex-illu* are the three possible entry points to process barcoded fastq files (blue squared blocks). In most setups (plain arrows), clipped or demultiplexed fastq files are mapped to the genome (grey squared block) using your favorite mapper and filtered for duplicate reads by the Je’s *markdupes* module using extracted UMIs. In more complex barcoding designs (e.g. composite barcodes, Supplementary Text), additional clipping before or after the sample demultiplexing step could be required (dashed arrows)

Currently available tools do not offer the flexibility required to process these different barcoding configurations and perform duplicate filtering using UMIs. Here we present Je, a suite of tools that can demultiplex fastq files (accommodating all described situations above), extract UMIs from demultiplexed files and filter (or flag) read duplicates taking UMIs into account (Fig. 2).

## Implementation

Je is implemented in Java 7 and uses the *htsjdk* (http://samtools.github.io/htsjdk/) and *picard* [8] libraries. Je has been designed with extensibility in mind with each sub-module (*demultiplex*, *demultiplex-illu*, *clip* or *markdupes*) encapsulated in its own package. This is reflected on the command line level where the command to run (*demultiplex*, *demultiplex-illu*, *clip* or *markdupes*) should be specified right after the *je* executable followed by relevant module’s options e.g. *je demultiplex < options>*, where *< options >* is the option list. The top level class *Je.java* is responsible to parse this command line and invoke the appropriate sub-module’s class (for example *Jeclipper.java* in the *jeclipper* package) with user’s provided options. The sub-module class is then responsible to validate user’s options before computing.

### The *demultiplex* command

The *demultiplex* command is used when the sample-encoding barcode is found at the beginning of the read (Fig. 1a, right). It can deal with SE and PE reads having barcodes in one or both reads, with or without UMIs (Additional file 1: Supplementary Text). This includes situations where barcodes contain degenerate positions (like in the individual-nucleotide resolution Cross-Linking and ImmunoPrecipitation (iCLIP) protocol), are combined with UMIs into composite barcodes (Fig. 1d, bottom) or found in different reads (e.g. sample-encoding barcode in read_1 and UMIs in read_2, Fig. 1c). Je’s *demultiplex* module offers many options to tune sample identification stringency (e.g. mismatch number, barcode combination), read processing (e.g. trimming, clipping) and output format (gzip compression, md5 checksum generation). In all situations that include UMIs (or degenerate barcodes), *demultiplex* output is fully compatible with Je’s *markdupes* command.

### The *demultiplex-illu* command

The *demultiplex-illu* command is used when sample-encoding barcodes are provided in separate fastq file(s) and UMIs are found at the beginning of the read(s). While CASAVA’s bcl2fastq2 tool is usually used to convert bcl files to fastq files and perform demultiplexing at the same time; it can also generate non-demultiplexed fastq files together with associated fastq index files (Fig. 1a, left). This alternative proves useful when debugging new protocols that use the index position for other purposes than sample encoding; or to overcome bcl2fastq2 barcode matching limitations (e.g. only allows up to two mismatches). Je’s *demultiplex-illu* module offers the same options as the *demultiplex* module and its output is fully compatible with Je’s *markdupes* command.

### The *clip* command

The *clip* command is used to extract UMIs from fastq files that do not require sample demultiplexing at the same time. Similarly to *demultiplex* and *demultiplex-illu* commands, extracted UMIs are added to the read headers (as expected by *markdupes*) and read headers are reformatted to fulfill read mappers requirements (most read mappers expect headers for read_1 and read_2 to be strictly identical). The *clip* module offers identical read processing (e.g. trimming, clipping) and output formatting options as the demultiplexing modules.

### The *markdupes* command

The *markdupes* command extends the popular Picard’s MarkDuplicates tool [8] by adding support for UMIs embedded in read headers (as generated by the *demultiplex*, *demultiplex-illu* or *clip* commands). This module takes mapped reads as input (in SAM/BAM format) and identifies PCR (and optical) read duplicates based on their mapping positions and UMIs. In short, reads identified as duplicates based on their mapping locations are further regrouped based on their UMIs (Additional file 1: Supplementary Text). All reads of a UMI group are declared duplicates but one (according to the chosen scoring strategy). Finally, duplicate reads are either discarded or included in output (with bitwise flag 1024). Je’s *markdupes* module supports random UMIs (any combination of a k-mer can occur) or runs with a predefined list of UMIs (as in e.g. NEXTflex™ kit from Bioo Scientific). In both situations, different options (in addition to all native Picard’s MarkDuplicates options) are offered to tune UMI comparison stringency like the number of mismatches to still consider two UMIs identical, or how to handle Ns found in UMIs.

### Galaxy integration

A wrapper for integration in Galaxy [9] was written for each Je sub-module following Galaxy guidelines and best practices. All wrappers (and Je code) were uploaded to the Galaxy toolshed [10] as a repository suite, enabling Galaxy administrators to either install each sub-module separately or together as a suite.

## Results and discussion

### Using UMIs significantly increases the number of useable reads

scRNA-seq is a powerful tool to quantify the extent of gene expression variability amongst a population of cells and, for example, reveal sub-populations of cells or new cell types. The low amount of starting material (combined with the low efficiency of RNA capture and cDNA synthesis) and the bias introduced by the substantial amplification required have been identified as major limitations and generally result in a high level of technical noise [7, 11]. By eliminating the noise introduced at the amplification step, the use of UMIs was demonstrated to be critical towards an accurate and absolute quantification of the number of original RNA messenger molecules present per cell [4, 12]; and globally facilitates distinguishing true biological variability from technical variability [7]. Generally, scRNA-seq data has a very high level of duplicate reads (as identified solely by their identical mapping position). Therefore, increasing the number of unique reads available for gene expression quantification is key, in particular for lowly to moderately expressed genes [11]. To quantify the gain of tagging reads with UMIs that are processed using the Je suite, we reprocessed 50 scRNA-seq single cell experiments from Islam et al. [12] (Additional file 1: Supplementary Methods) and identified unique reads with and without taking the UMIs into account (using *je markdupes* and Picard MarkDuplicates [8], respectively); which, in this case, directly translates to the number of RNA molecules present in the cell. We quantified the gain obtained using the UMIs as the number of duplicate reads reassigned as unique reads once the UMIs were taken into account. Expressed as a percentage relative to the number of unique reads identified without accounting for the UMIs, the gains ranged from 13 to 36 % with an average of 24 % (Additional file 1: Figure S1).

iCLIP also suffers high duplication rates due to the low number of biologically relevant genomic positions. To evaluate the impact of using UMIs for this type of experimental data, we analyzed iCLIP human samples published by Zarnack et al. [13] (Additional file 1: Supplementary Methods) and observed gains in the number of useable reads ranging from 10 to 36 % with an average of 21 % (Additional file 1: Figure S2).

The advantage of using UMIs is not limited to scRNA-seq or iCLIP experiments. Indeed, duplicate read filtering (using e.g. Picard MarkDuplicates) is standard practice in the processing of DNase I hypersensitive sites sequencing (DNAse-seq) and chromatin immunoprecipitation sequencing (ChIP-seq) data, which in single-end sequencing results in capping the coverage (number of reads or fragments overlapping a specific genomic position). As the sequencing depth increases, this approach severely impacts the signal-to-noise ratio as the background coverage increases while the signal coverage reached its upper limit. A straightforward solution is to systematically introduce UMIs and use Je to uniquely identify fragments in DNAse-seq and ChIP-seq libraries to avoid an artificial limitation of the dynamic range.

### Je offers a unique set of features

Although a number of demultiplexing tools have been published, Je comes with a unique set of features when compared to available tools (Additional file 1: Table S1). For instance, deML [14] and bayexer [15] focus on improving Illumina TruSeq indices demultiplexing in the particular situation of low quality reads, TagGD [16], GBSX [17] and FLEXBAR [18] specialize in barcode design and provide debarcoding algorithms able to handle barcodes of variable length or found at variable position in the read, while fastq-multx [3] and fastx_barcode_splitter [2] only accommodate the standard in-line barcoding approaches (barcodes found at reads start and of fixed length). Although some of these tools might be more suited than Je in particular situations, none of them offer UMI support and should therefore be combined with specialized tools such as UMI-tools [19] or Je (*clip* and *markdupes*) when reads contain UMIs. Similarly, modules from MIGEC [20], a suite of tools specialized in the processing of T-cell receptor repertoire sequencing (RepSeq) data, can demultiplex and utilize UMI-tagged data but with a fundamental different deduplication approach in that it directly works on non-aligned reads and assembles them into consensus sequences. In addition, MIGEC (like UMI-tools) cannot accommodate for predefined list of UMIs. Taken together, we believe that the extent and flexible nature of the features offered by Je are unique, and constitute a valuable suite for data with complex experimental designs.

## Conclusions

Je offers the necessary tools to address most barcoding situations with and without UMIs (also see Additional file 1: Supplementary Text) and the identification of PCR duplicates based on extracted UMIs. In standard experimental set ups (one barcode per sample, identical barcodes at both fragments’ ends) and using equivalent options (i.e. mismatch number), Je *demultiplex* produced identical results when compared to other demultiplexing tools [2, 3] and performed 3.8 times faster and 4.5 times slower than the popular FASTX [2] (barcode_splitter) and eautils [3] (fastq-multx) packages, respectively (Additional file 1: Supplementary Methods). However, Je *demultiplex* and *demultiplex-illu* can handle more complex designs such as mixing samples encoding barcodes and UMIs. Using Je to process complex public scRNA-seq and iCLIP data that leverage the advantages of UMIs, we observed an increase of unique reads up to 36 % when compared to Picard MarkDuplicates [8], which cannot account for the presence of UMIs.

To broaden Je accessibility, we developed wrappers for Galaxy [9] and made Je available through the Galaxy toolshed [10].

## Additional files

Additional file 1: Supplementary Text. Installation notes, Je usage details to address simple and advanced barcoding configuration with or without UMIs. **Supplementary Methods.** Description of the scRNA-seq and iCLIP data analysis. **Figure S1.** Impact of using UMIs in single-cell RNA-seq experiments. **Figure S2.** Impact of using composite barcodes in iCLIP experiments. **Table S1.** Comparison of diverse demultiplexing tools. (DOCX 254 kb)
Additional file 2: Archive containing an executable Jar and a wrapper script. (ZIP 9774 kb)

## Abbreviations

ChIP-seq: chromatin immunoprecipitation sequencing
DNAse-seq: DNase I hypersensitive sites sequencing
iCLIP: Individual-nucleotide resolution UV CrossLinking and Immunoprecipitation
NGS: Next generation sequencing
PCR: Polymerase Chain Reaction
PE: Paired end
scRNA-seq: Single-cell RNA sequencing
SE: Single end
UM: Unique Molecular Identifier
