# Supplementary material for: Je, a versatile suite to handle multiplexed NGS libraries with unique molecular identifiers
Source: BMC Bioinformatics. 2016 Oct 8;17:419. doi: 10.1186/s12859-016-1284-2 (PMC5055726; doi:10.1186/s12859-016-1284-2)
Supplement: Additional file 1: Supplementary Text. — Installation notes, Je usage details to address simple and advanced barcoding configuration with or without UMIs. Supplementary Methods. Description of the scRNA-seq and iCLIP data analysis. Figure S1. Impact of using UMIs in single-cell RNA-seq experiments. Figure S2. Impact of using composite barcodes in iCLIP experiments. Table S1. Comparison of diverse demultiplexing tools. (DOCX 254 kb) [file 12859_2016_1284_MOESM1_ESM.docx]

**Additional file 1**

**Supplementary Text**

# Forenotes / Installation

Code is open source and is available at http://gbcs.embl.de/je

For convenience, we also release an executable jar (version 1.0) and its shell script companion as supplements. To execute *Je*, simply place these two files in the same folder and make sure that Java 7 or higher is available on your computer. Assuming you have placed files in the “~/je-suite/” directory, calling:

> ~/je-suite/je

will print the command line help

# Demultiplexing paired-end FASTQ FILES (*demultiplex* module)

When following a protocol without specific index primers (Supp. Fig. 1A, right), the position of barcodes are specified using the *Je* BPOS option (Supp. Fig. 1B). In case barcodes are found at both ends (Supp. Fig. 1C), then either both barcodes encode the same information and any of the extracted barcoding sequences could be used for sample lookup (Supp. Fig. 1C case 1, BRED=true); or they encode different information and the user must specify which one(s) to use for sample lookup (Supp. Fig. 1C case 2, BRED=false).

In the first scenario (BM=BOTH, BRED=true), *Je* can combine both barcoding sequences in a strict fashion (S=true), so that the read pair is kept only if both barcoding sequences resolve to the same sample; or in a loose (S=false) fashion in which a read pair is discarded only when the barcoding sequences resolve to different samples. Below is a command line example with the loose combination option:

> je demultiplex F1=fwd.fastq F2=rev.fastq BF=codes.txt BPOS=BOTH BM=BOTH BRED=true S=false

Because these settings are the default ones, the command line is as simple as:

> je demultiplex F1=fwd.fastq F2=rev.fastq BF=codes.txt

In the second scenario (Supp. Fig. 1C case 2), *Je* requires information whether both barcode sequences (BM=BOTH) are needed for sample identification (i.e. each sample is barcoded with a pair of barcodes) or if only one of them encodes the sample information (e.g. BM=READ_1), while the second is a UMI used for PCR duplicate identification. Below is a command line example for files with a 6 bp sample-encoding barcode in read_1 and a 8 bp long UMI in read_2 :

> je demultiplex F1=fwd.fastq F2=rev.fastq BF=codes.txt BPOS=BOTH BM=READ_1 LEN=6:8

# Advanced barcoding scenarios (*demultiplex* and *demultiplex-illu* commands)

Barcodes are provided as a simple tab-delimited file featuring the sample name (first column) and the barcode (second column). The barcodes are specified with the ‘A’, ’C’, ‘G’, ‘T’, ‘N’, ‘:’ and ‘|’ characters. The ‘|’ character is used as a barcode separator (logical OR) to associate one sample with multiple barcodes. The ‘:’ character is used to differentiate barcode(s) applicable to the first read (*READ_1*) from the one(s) applicable to the paired read (*READ_2*). Finally, ‘N’ indicates positions to ignore during sample lookup, for example when processing iCLIP(Konig *et al.*, 2011) reads or composite barcodes(Shiroguchi *et al.*, 2012).

## Simple barcode file example

This reflects the usual situation i.e. each sample is identified by one barcode only.

sample1 GATGCT

sample2 CCGTAT

In paired end (PE) sequencing, the above barcode file would be interpreted as follows:

- When using *demultiplex*, the barcode is expected to be the same in both read (default) or found in one of the two reads only (BM option).
- When using *demulitplex-illu* (with two index files), the barcode is expected to be the same in both index files.

## Specifying multiple barcodes per sample with the | (OR) character

sample1 GATGCT|TTGCGG

sample2 CCGTAT|CGTACG|GGAGGA

Note that the number of barcodes associated to each sample can be different.

## Specifying different barcodes per read (PE only)

The following example is common when using Illumina dual indexing.

To specify that ‘sample1’ is indexed with GATGCT in *READ_1* and TTGCGG in *READ_2*, and ‘sample2’ is indexed with CCGTAT in *READ_1* and CGTACG in *READ_2*; the following barcode file can be assembled:

sample1 GATGCT:TTGCGG

sample2 CCGTAT:CGTACG

Also note that one could still specify multiple barcodes using the | (OR) character:

sample1 GATGCT|CCGTAT:TTGCGG|CGTACG

## Specifying degenerate positions in barcodes using N (*demultiplex* only)

Some protocols (e.g. iCLIP) use barcodes containing degenerate positions. In such a situation, all positions that belong to the barcode must be specified to let *demultiplex*:

1. understand the exact length of the barcode and extract the correct sequence and,
2. use only the relevant positions (i.e. ignore the ones marked N) when matching the read sequence to the expected barcodes.

sample1 NNNCCGGNN

sample2 NNNTTAGNN

Importantly, when barcodes contain Ns, *demultiplex* will append the actual extracted read sequence to the read header as these nucleotides can later be used as UMIs to flag duplicate reads (e.g. NCCTNN could become TCCTGC and ACCTAT for two different reads that map at the same genomic location). For the same reason, it is important to specify all the trailing Ns as in the example above. While the same demultiplexing results would be obtained by adding the option XT=2 (which means ‘clip 2 extra bases after barcode’) to the command line and the barcode file below, *markdupes* would obviously produce different results due to the different UMIs produced (9 bases long UMIs with 5 Ns in the first case versus 7 bases long UMIs with 3 Ns in the second).

sample1 NNNCCGG

sample2 NNNTTAG

## Controlling output file names and location using the barcode file

In all situations described above, a third column (single end (SE) case) or a third and fourth (PE case) column could be added in the barcode file to individually specify output file names. When file names (without path) are given, all output files are created in the ‘output dir’ (*O* option). When file paths are given (see below for PE examples), the location of each output file can be individually controlled.

sample1 GATGCT sample1_fwd.fastq.gz sample1_rev.fastq.gz

sample2 CCGTAT sample2_fwd.fastq.gz sample2_rev.fastq.gz

or to control also the paths :

sample1 GATGCT /exp1/sample1_fwd.fastq.gz /exp1/sample1_rev.fastq.gz

sample2 CCGTAT /exp2/sample2_fwd.fastq.gz /exp2/sample2_rev.fastq.gz

# composite barcodes - sample-encoding WITH UMIs at same Read end (*demultiplex* only)

Figure1D, bottom: Combine UMIs with a composite barcode.

There are two options to deal with this scenario.

## First option

The first option is to define a barcode with degenerate positions using Ns, as explained in the previous section.

When the UMIs are found before the sample-encoding barcode:

sample1 NNNNNNCCGGAA

sample2 NNNNNNTTAGCG

Or when the UMIs are found after the sample-encoding barcode:

sample1 CCGGAANNNNNN

sample2 TTAGCGNNNNNN

Importantly, this option results in extracting the 12 bases as a unique barcode i.e. the read name would hold these 12 bases and Je’s *markdupes* module will then use these 12 bases as UMIs. This is normally not an issue for *markdupes* unless you run *markdupes* with a pre-defined list of molecular barcodes. In such a situation, you should make sure that each predefined UMI also contains the sample-encoding barcode.

## Second option

An alternative is to combine *demultiplex* and *demultiplex-illu* with the *clip* module:

1. When UMIs are found before the sample-encoding barcode, *clip* is run before *demultiplex* to extract the UMIs.
2. When UMIs are found after the sample-encoding barcode, *demulitplex* is run first followed by *clip* to extract the UMIs

In both cases, the sample barcode file is the same and does not specify the UMIs:

sample1 CCGGAA

sample2 TTAGCG

# Combining Illumina indexing with molecule tagging (*demultiplex-illu only*)

Figure1A, left: Illumina TruSeq™ protocol.

When specific index primers are used, sample-encoding barcodes are provided in separate fastq files (I1 and I2 options). In this situation UMIs are found at the beginning of READ_1 and/or READ_2 and the BPOS option indicates in which read(s) UMIs are present (Supp. Fig. 1D, top).

# Additional features available in *demultiplex*, *demultiplex-illu* and *clip* commands

*Je* offers additional options for read trimming:

- XT and ZT to trim an arbitrary number of bases from the 5’ or 3’ read ends, respectively
- GZ: for compression of the output files.
- MD5: to generate a md5 checksum file for each output file at no extra I/O cost.

In specific Paired End situations (e.g. Supp. Fig. 1C case 2), relevant options (MM, MMD, Q, XT, ZT) accept two distinct values (e.g. “ZT=0:6”). With this notation, different values will be applied to read_1 and read_2. For example, if a 6-base barcode is only found in the read_1 (Supp. Fig. 1B, BPOS=READ_1), then read_2 can be specifically trimmed by 6 bases at its 3’ end with ZT=0:6. The reads will then be of identical lengths.

# Identifying DUPLICATES WITH Je *markdupes*

## *Je*’s *markdupes* uses the UMI information that *demultiplex*, *demultiplex-illu* or *clip* extracted from read sequences and embedded in the read names.

## Default command line

> je markdupes I=sample.bam O=sample-nodupes.bam M=markdupes_stats.txt MM=2 REMOVE_DUPLICATES=true

In this command line, the only *Je* *markdupes* specific option is “MM=2”, specifying the maximum number of mismatches to consider two UMIs identical. Other options are native options of Picard’s MarkDuplicates.

## Indicating where UMIs are located in read names (SLOTS option)

In fastq read headers, the ‘:’ is the usual delimiter for meta-data, see below for an example. By default, *markdupes* tries to find the UMIs at the last position in the header. This default sometimes needs to be adapted to match the experimental set-up (e.g. presence of two UMIs per read) and/or the location of the UMIs in the read name. This is achieved with the SLOTS option that can be specified in the command line as many times as there are UMIs present in the read name.

Consider the following read name that holds 3 different barcodes in the last three fields (fields are delimited by ‘:’)

HISEQ:44:C6KC0ANXX:8:2112:20670:79594:CGATGTTT:GATCCTAG:AAGGTACG

By default, only AAGGTACG would be considered a UMI, to indicate that

- the three barcodes are UMIs, add “SLOTS=-1 SLOTS=-2 SLOTS=-3” in the command line
- the 2 last barcodes are UMIs, add “SLOTS=-1 SLOTS=-2”

Note that *markdupes* understands counting from the end using negative numbers and counting from the start with positive numbers, starting from 1.

## Understanding duplicate identification procedure

All reads mapping at the same location are first identified using the native Picard’s MarkDuplicates procedure. For each read, the UMIs are then extracted from the read name and used to decide whether two reads originate from different biomolecules (UMIs are different) or from PCR amplification (UMIs are identical). This last step is different given that a predefined list of expected UMIs is known or not.

## Procedure when running with a predefined list of UMIs

When UMIs are known in advanced (e.g. using kits like the NEXTflex™ kit from Biooscientific), *markdupes* proceeds as follows to process a group of reads with an identical genomic location:

1. For each read, check each extracted UMI for the number of N it contains
   1. if this number is greater than MAX_NUMBER_OF_N option, place the read into a special ‘undef’ read group, and proceed to next read
   2. else go to next step
2. Compare each extracted UMI to the pre-defined list of UMIs allowing for a maximum of MISMATCHES (MM) i.e. the first predefined UMI that is not more than the number of mismatches different from the UMI is considered as the successful match. Note that Ns are *not* considered as mismatches, as that would artificially inflate the number of different UMIs.
3. Concatenate each identified UMI into a unique “UMI_KEY”.
4. Regroup all reads by their UMI_KEY (the ‘undef’ read group is also considered as a UMI_KEY)
5. For each UMI_KEY group,
   1. find the best read according to the native Picard’s MarkDuplicates duplicate scoring strategy (DUPLICATE_SCORING_STRATEGY option)
   2. flag all reads but the best one as duplicates
6. Proceed to the next group of reads with identical mapping location

## Running without a predefined list of UMIs

When UMIs are not known in advance, *markdupes* proceeds as follows to process a group of reads with identical genomic location:

First Phase: regroup reads based on their combined UMIs

1. For each read, concatenate UMIs into a unique UMI_KEY and check the number of N in UMI_KEY
   1. if this number is greater than MAX_NUMBER_OF_N option, place the read into a special ‘undef’ read group,
   2. go to next read
2. Regroup all reads (not in the ‘undef’ group) based on their UMI_KEY and sort these read groups (1) by the number of Ns their UMI_KEY contain (least Ns first) and, for identical number of Ns, (2) by the number of reads found in the read group (with highly populated groups first groups).
3. Let’s call this list of sorted groups G and let’s call an individual read group G_i_ with *i* indicating its ordered position in G (0 being the best).

Second Phase: merge read groups with similar UMI keys

1. Create R, an ordered list to hold final read groups
2. Initialize R with R_0_, a copy of G_0_. R_0_ is uniquely identified by a set of unique aliases (A_0_) that first contains the UMI_KEY of G_0_ as unique alias
3. Iterate over G (following sorting order and starting at 1) and for each group G_i_:
   1. Consider each R_i_ iteratively (R_0_ first, R_1_ second…)
   2. For each R_i_, compare G_i_‘s UMI_KEY to R_i_ aliases allowing for a maximum of MISMATCHES (MM) mismatches. Note that Ns are *not* considered as mismatches.
      1. If UMI_KEY of G_i_ matches any of R_i_‘s aliases, add all G_i_‘s reads in R_i_ and add G_i_‘s UMI_KEY to the alias list of R_i_
      2. If UMI_KEY of G_i_ does not match any of R_i_‘s aliases, add G_i_ as a new group in R (and initialize its alias set with the UMI_KEY of G_i_)
4. Finally, for each read group R_i_ of R and also for the ‘undef’ group,
   1. find the best read according to the native Picard’s MarkDuplicates duplicate scoring strategy (DUPLICATE_SCORING_STRATEGY option)
   2. flag all reads but the best one as duplicates

**Supplementary Methods**

# demultiplexing time Comparison

Running times were obtained on a Dell R910 server (40 Intel Xeon CPU E7- 4870 at 2.40GHz cores) using a multiplexed (10 samples) gzipped fastq file containing 153818656 reads sequenced on HiSeq2000 in single end mode (51-bases long) using the following command lines (1 mismatch allowed).

Fastx_barcode_splitter (fastx_toolkit version 0.0.13.2) completed in ~68 minutes when ran with the following command:

zcat reads.txt.gz | fastx_barcode_splitter.pl --bcfile barcodes.txt --prefix FASTX --bol --mismatches 1

Fastq-multx (version ea-utils.1.1.2-537) completed in ~4 minutes when ran with the following command:

fastq-multx -m 1 -d 1 -B barcodes.txt reads.txt.gz -o r1.%.fq

Je completed in ~18 minutes when ran with the following command:

je demultiplex F1=reads.txt.gz BF=barcodes.txt

# ANalysis of single-cell RNA-seq

We obtained single-cell RNA-seq data[10] from <ftp://ftp-trace.ncbi.nlm.nih.gov/sra/sra-instant/reads/ByStudy/sra/SRP/SRP022/SRP022764> and analysed data for 50 cells (accession numbers from SRR1043434 to SRR1043484). SRA files were converted to fastq format using fastq-dump (sra_toolkit v2.5.4). According to the authors, each read is expected to start with a 5 bp unique molecular identifier (UMI), followed by 3-5 guanines, followed by the 5’ end of the transcript. Je-clip (Galaxy Tool Version 1.0) was used to extract and add the 5-bases UMI to the read name, the next 5 bases were additionally trimmed (XTRIM=5 option). Resulting fastq files were mapped to the mouse genome (mm10 version) in Galaxy using Tophat Gapped-read mapper for RNA-seq data (Galaxy Tool Version 0.7) and default settings. Multi-mapping reads were eliminated using the bamtools_filter (Galaxy Tool Version 0.0.1) requesting reads to be primarily mapped and flagged with a NH:i:1 tag. Finally, duplicate reads were identified using the Picard MarkDuplicates (Galaxy Tool Version 1.126.0) and Je-MarkDuplicates (Galaxy Tool Version 1.0 of je markdupes) allowing for a maximum of 1 mismatch and 1 N per UMI.

For each sample *i*, the number of unique reads (mapped reads minus duplicate reads) UP_i_ and UJ_i_ were computed from the reports generated by Picard MarkDuplicates and Je-MarkDuplicates, respectively. Finally, the gain (as reported in Figure S1 and main text) in unique reads (UJ_i_ - UP_i_) was expressed as a percentage relative to UP_i_ i.e. G_i_ = (UJ_i_ - UP_i_) / UP_i_ * 100.

# ANalysis of iCLIP-seq

Fastq files were obtained directly from the authors[11]. Original reads contain composite barcodes made of 3 random bases followed by 4 specific bases encoding the sample identity and 2 additional random bases before RNA sequence starts (e.g. NNNTTGTNN). The original fastq files were demultiplexed using *je demultiplex* (default parameters) which resulted in fastq files holding 9 bases long tags in the read headers (of which 5 bases are variable) that can be regarded as UMIs. The demultiplexed fastq files were trimmed for adapters using cutadapt v1.9.1 (with option -m 15). Reads were mapped on the human genome (GRCh37_Ensembl75) using STAR version STAR_2.5.0a and options --outFilterMismatchNmax 3 --outFilterMismatchNoverLmax 0.12 --alignEndsType EndToEnd. Multi-mapping reads were eliminated using the bamtools_filter (Galaxy Tool Version 0.0.1) requesting reads to be flagged with a NH:i:1 tag. Finally, duplicate reads were identified using the Picard MarkDuplicates (Galaxy Tool Version 1.126.0) and Je-MarkDuplicates (Galaxy Tool Version 1.0 of je markdupes) allowing for a maximum of 1 mismatch and 1 N per UMI.

For each sample *i*, the number of unique reads (mapped reads minus duplicate reads) UP_i_ and UJ_i_ were computed from the reports generated by Picard MarkDuplicates and Je-MarkDuplicates, respectively. Finally, the gain (as reported in Figure S2 and main text) in unique reads (UJ_i_ - UP_i_) was expressed as a percentage relative to UP_i_ i.e. G_i_ = (UJ_i_ - UP_i_) / UP_i_ * 100.

**Supplementary Figures**

**Figure S1. Impact of using UMIs in single cell RNA-seq**. Duplicate reads were identified in 50 single cell RNA-seq samples from Islam *et al*[10]. The plot shows the number of number of duplicate reads reassigned as unique reads once UMIs are taken into account (as reported by Je- MarkDuplicates). This number is expressed as a percentage relative to the number of unique reads initially identified by Picard MarkDuplicates (i.e. when UMIs are not considered). The gain in unique reads ranges from 13% to 36% with an average of 24%.

**Figure S2. Impact of using composite barcodes as UMIs in iCLIP-seq**. Duplicate reads were identified in 7 iCLIP samples from Zarnack *et al*[11]. The plot shows the number of duplicate reads reassigned as unique reads once UMIs are taken into account (as reported by Je- MarkDuplicates). This number is expressed as a percentage relative to the number of unique reads initially identified by Picard MarkDuplicates (i.e. when UMIs are not considered). The gain in unique reads ranges from 10% to 36% with an average of 21%

|  | **Je** | **deML** | **bayexer** | **TagGD** | **GBSX** | **FLEXBAR** | **FASTX Barcode splitter** | **fastq-multx (ea-utils)** | **migec** | **UMI-tools** |
| --- | --- | --- | --- | --- | --- | --- | --- | --- | --- | --- |
| ***Demultiplexing:*** |  |  |  |  |  |  |  |  |  |  |
| **In-line barcode** | ✓ | ✗ | ✗ | ✓ | ✓ | ✓ | ✓ | ✓ | ✓ | - |
| **Illumina Indices** | ✓ | ✓ | ✓ | ✗ | ✗ | ✓ | ✗ | ✗ | ✗ | - |
| **Color Space** | ✗ | ✗ | ✗ | ✗ | ✗ | ✓ | ✗ | ✗ | ✗ | - |
| **Trimming** | ✓ | ✗ | ✗ | ✗ | ✗ | ✓ | ✗ | ✓ | ✓ | - |
| **Allow mismatches in barcodes** | ✓ | ✓ | ✓ | ✓ | ✓ | ✓ | ✓ | ✓ | ✓ | - |
| **Composite Barcode**  **(in-line)** | ✓ | - | - | ✗ | ✗ | ✗ | ✗ | ✗ | ✓ | - |
| **Barcode position**  **(in-line)** | At start | - | - | Any | Within first 2 bases | Any | At start/end | At start/end | Any | - |
| **Variable barcode length**  **(in-line)** | ✗ | - | - | ✗ | ✓ | ✗ | ✗ | ✗ | ✓ | - |
| **Paired-End (PE)** | ✓ | ✓ | ✓ | ✓ | ✓ | ✓ | ✗ | ✓ | ✓ | ✓ |
| **Different barcode per read (PE in-line)** | ✓ | - | - | ✗ | ✓ | ✓ | ✗ | ✓ | ✓ | - |
| **Barcode Identification** | Hamming | Maximum Likelihood | naïve Bayes classifier | Levenshtein | Hamming, Knuth, indelmis, misindel | Needleman-Wunsch | Hamming | Hamming | Exact Seed Match | Hamming |
| ***UMI Support:*** |  |  |  |  |  |  |  |  |  |  |
| **UMI detection** | ✓ | ✗ | ✗ | ✗ | ✗ | ✗ | ✗ | ✗ | ✓ | ✓ |
| **Random UMI-based duplicate filtering** | ✓ | - | - | - | - | - | - | - | ✓ | ✓ |
| **Predefined UMI-based duplicate filtering** | ✓ | - | - | - | - | - | - | - | ✗ | ✗ |
| **Allow MM in UMI** | ✓ | - | - | - | - | - | - | - | ✓ | ✓ |
| ***Other:*** |  |  |  |  |  |  |  |  |  |  |
| **Barcode design** | ✗ | ✗ | ✗ | ✓ | ✓ | ✗ | ✗ | ✗ | ✗ | ✗ |
| **GUI** | Galaxy | ✗ | ✗ | ✗ | Galaxy | Galaxy | Galaxy | ✗ | ✗ | ✗ |

**Table S1.** **Comparison of features of different demultiplexing tools.** The marks ✓,✗ and – translate to “supported”, “not-supported” and “not applicable” respectively. ***Demultiplexing features***: support for in-line barcodes found within the read sequence (In-line barcode), support for Illumina TruSeq indexing (Illumina indices), support for barcodes encoded in color space (Color Space), support for in-line composite barcode (Composite Barcode (in-line)), ability to trim reads in addition to clip barcodes (Trimming), ability to handle mismatches in the barcode sequence (Allow mismatches in barcodes), support for in-line barcode positions within the read (Barcode position (in-line)), support for in-line barcodes of variable length (Variable barcode length(in-line)), support for paired-end sequencing (Paired-End (PE)), ability to handle a different in-line barcode per read in paired-end (Different barcode per read (PE in-line)) and method(s) used to compare read sequence with barcode (Barcode Identification). ***UMI support features***: ability to extract UMIs from reads (UMI detection), support for UMI-based duplicate filtering using random UMIs (Random UMI-based duplicate filtering) and/or using a pre-defined list of UMIs (Predefined UMI-based duplicate filtering) and the ability to handle mismatches in the UMI sequence during filtering (Allow MM in UMI). ***Other features***: support for barcode design (Barcode design) and which graphical user interface is available(GUI).
